# Gendered racial disparities in health of parents with children with developmental disabilities

**DOI:** 10.3389/fpsyg.2022.926655

**Published:** 2022-09-02

**Authors:** Juha Lee, Manjing Gao, Chioun Lee

**Affiliations:** Department of Sociology, University of California, Riverside, Riverside, CA, United States

**Keywords:** race, gender, life course, caregiving, developmental disabilities, health disparities

## Abstract

**Background:**

There is little information on (1) how adverse experiences in early life are associated with the risk of having a child with health problems and (2) whether the health of racial and gender minority groups would be particularly compromised if they have developmentally disabled (DD) children.

**Objective:**

By integrating life-course perspectives and the intersectionality framework, we examine (1) the extent to which parents’ early-life adversities (ELAs) are associated with having children with DD or other health issues and (2) whether the association between having DD children and parental (physical and mental) health varies across race–gender groups after accounting for ELAs.

**Methods:**

Using data from Black and White parents from the Midlife in the US Study (*n* = 7,425; 18% Black), we employed (1) multinomial logistic regression models to investigate the degree to which ELAs are associated with parenting types (having a child with DD, a child with recent illness, or a child without these health issues) and (2) multiple regression models with a three-way interaction term to investigate whether the gender–parenting type association differs by race.

**Results:**

With more adversities, the probability of having children with health issues increases for all race–gender groups, but most dramatically for Black women. Having DD children is associated with more chronic illnesses and functional limitations for women than men, with the largest burden for Black women, yet neither gender nor racial differences in depressive symptoms. Our results highlight that while raising children with DD takes a toll on the health of all parents, the strain might be larger for Black mothers.

**Conclusion:**

The adverse effects of parenting a child with DD is more pronounced for Black women than for other race–gender groups indicating opportunities to promote community-based programs for these parents.

## Introduction

Having an ill or disabled family member is among the most stressful and taxing life events, often requiring individuals to become long-term caregivers (Vitaliano, 1997). In 2020, one in five US adults identified themselves as informal caregivers and the proportion is higher for women and racial minorities (AARP, 2020). Importantly, the consistent increase in the prevalence of U.S. children with developmental disabilities (DD) over the past two decades (Boyle et al., 2011; Zablotsky et al., 2019), for example 13.87% in 1997–2008 to 16.93% in 2009–2017, indicates that more parents will be responsible for caring for their children with DD even into midlife and old age.

Despite some positive aspects of parenting DD children (Beighton and Wills, 2017), numerous studies have identified psychological and physical costs of caring for DD children (Song et al., 2016, 2018; Namkung et al., 2018; Sloan et al., 2020). Yet, extant studies have paid little attention to how the association between having DD children and parents’ health can be confounded by early-life factors of parents. For example, adverse childhood experiences can influence both the risk of offspring’s developmental delays (Folger et al., 2018) and individuals’ health outcomes (Lee et al., 2017; Lee and Ryff, 2019). More rigorous work is needed that examines whether having DD children compromises parental health even after accounting for a wide array of early-life adversities (ELAs) experienced by parents. Moreover, there is a scarcity in the caregiving and aging literature on (1) racial disparities in caring for children with DD and parental toll in the context of health and (2) how such gendered strains of caregiving vary by race. By integrating life-course perspectives and the intersectionality framework, our study expands prior work on family caregiving and health.

## Background

### Gender disparities

Caring for children with DD may compromise mothers’ vs. fathers’ health differently. Despite changing trends in gender roles in unpaid household work (Bianchi et al., 2012), mothers are still expected to be the primary caregiver for their child, while fathers remain the main breadwinner for the family (Wheatley et al., 2018). Although some fathers with DD children show involvement and engagement in caregiving (Fox et al., 2015), mothers with DD children are more likely than fathers to cut working hours (Seltzer et al., 2001) and be more involved with hands-on caregiving responsibilities, which may result in more adverse effects on their health compared to fathers (e.g., Pinquart and Sörensen, 2006; Dunn et al., 2019). For example, in comparison to fathers with DD children, mothers who care for DD children are likely to report higher body mass index (BMI), lower self-rated health (Seltzer et al., 2001) and higher levels of physical symptoms (Namkung et al., 2018).

In terms of mental health, the effects of having a DD child are mixed and further examination of gender disparities is needed. Specifically, some studies show that mothers report poorer mental health than fathers (Olsson and Hwang, 2001) while other studies demonstrate no gender difference (e.g., Ha et al., 2008). Interestingly, some studies found that social and psychological factors might differently moderate the adverse impact of having a DD child. For example, Homan et al. (2020) reported that both fathers and mothers of a child with DD have higher levels of depressed affect and lower levels of positive affect than parents of children without DD. They also reported that having a high level of generativity mitigates negative affect for mothers, but not for fathers. Olsson and Hwang (2006) found that, among mothers and fathers with DD children, working less than 20 h per week is associated with low well-being; working more than 20 h per week is associated with higher well-being for fathers only.

### Racial disparities

The literature on family caregiving in general shows that the consequences of caregiving vary by race. Using meta-analysis, Pinquart and Sörensen (2005) concluded that most ethnic minority caregivers have worse physical health but better mental health, for example, lower levels of depression and more perceived uplifts. Racial disparities in resources and coping mechanisms—care provision, cognitive coping strategies, and psychological and social assets—might play a major role in shaping racial patterns in physical vs. mental health outcomes of caregiving. That is, lack of financial resources and limited access to quality healthcare may exacerbate physical burden among Black caregivers (Magaña et al., 2012). Black caregivers, however, tend to report higher levels of informal coping resources rooted in strong familism and religiosity (Mouzon, 2017; Brown et al., 2020) and greater rewards from caregiving (Liu et al., 2021).

It is an open question whether racial patterns in family caregiving—worse physical health, but better mental health for Black caregivers—hold for the health of parents with DD children. In comparing Black parents with vs. without DD children, studies have reported that having DD children does not compromise mental health. For example, Magaña and Smith (2006) found that Black mothers caring for adult children with mental illness tend to suffer from physical illness yet are more resilient in managing their emotions. Similarly, among Black parents, having DD children is associated with elevated number of somatic symptoms but not with compromised affect (Ha et al., 2011). In comparing White vs. Black parents, Pruchno et al. (1997) found that compared to White parents, Black parents are likely to have fewer resources for caregiving, yet they are less likely to evaluate their caregiving role negatively and more likely to report better mental health and subjective well-being. Similarly, Picket et al. (1993), testing Black and White parents who have children with severe mental illness, found that Black caregivers report higher levels of self-esteem and lower levels of depressive symptoms than White caregivers. These findings suggest that Black parents with DD children might have more resilient mental health, but not physical health, than their White counterparts. However, prior studies used small community-based samples (e.g., Pruchno et al., 1997) or focused on the impact of having a DD child within a racial group (e.g., Ha et al., 2011).

### The role of parents’ early-life factors

The life-course perspective posits that individuals’ cumulative past experiences from varying life domains may influence future outcomes (Settersten, 2003). An individual’s early-life experiences, in particular, are precursors to later health consequences (Kuh et al., 2003; Nelson et al., 2020). Many empirical studies have shown that adverse childhood experiences (or ELAs) lead to neuropsychiatric outcomes including major depressive disorder and anxiety disorder (Widom et al., 1999; Scott et al., 2010) and increase risk for the leading causes of death in adults such as obesity, cardiovascular disease, and cancer (Felitti et al., 1998). Recent studies have addressed the extent to which ELAs influence the next generation’s lifestyles and health (McDonnell and Valentino, 2016; Folger et al., 2018). A growing body of studies, indeed, shows the significant role of parental, particularly maternal, experiences in early life—for example, harmful environment, unhealthy lifestyles, and adverse health conditions—on offspring’s health conditions, including developmental process (Sun et al., 2017; Folger et al., 2018) and physical health and emotional problems (Lê-Scherban et al., 2018). For example, experiencing ELAs is adversely associated with reproductive health and the health of offspring, as evidenced by a short gestation period and low birthweight in infants (McDonnell and Valentino, 2016).

There is a wide array of genetic, environmental and social factors that are associated with the risk of having a DD child. Prior studies suggest that poverty and its related stress, and risk behaviors are associated with potential risk factors of developmental delays (Blair and Raver, 2012), for example, alcohol and tobacco use during pregnancy (Herrmann et al., 2008), infections during pregnancy, and hazardous and toxic living environments during offspring’s childhood [Centers for Disease Control and Prevention (CDC), 2020]. Given that Black children and youths are more likely than their White counterparts to be exposed to impoverished and stressful environments (Giano et al., 2020) and have fewer resources throughout the life span to compensate for these adverse early-life exposures (Ferraro and Shippee, 2009), one can speculate that ELAs might disproportionally affect Blacks in terms of their risk of having DD offspring as well as later-life health problems. Yet, few studies have examined the role of ELAs in explaining racial disparities in the risk of having DD children and the health of parents with DD children.

### Aims of the current study

We investigate whether the health of racial and gender minority groups would be particularly compromised if they have DD children. Given gender disparities in caregiving responsibilities, we hypothesize that having DD children compromises health more for women than men. Black women, in particular, experience unique forms of oppression and deprivation derived from both racism and sexism (Collins, 1986; Pager and Shepherd, 2008; Thomas et al., 2008). Thus, we expect that the adverse effects of parenting a child with DD will be more pronounced for Black women than for other race–gender groups, particularly in the realm of physical health outcomes. Finally, racial minorities and women are more likely to experience multiple ELAs, and the adverse impacts of ELAs on later-life health are well-known. Therefore, we view ELAs as possibly important life-course confounders of racial and gender disparities in the association between having DD children and parental health. We expect that individuals, particularly racial minority mothers who experienced ELAs, are more likely to have DD children. Controlling for ELAs will reduce the effects of having DD children on parental health outcomes.

## Data and methods

### Sample

Data come from the MIDUS study, a national survey of health and aging. Respondents—English-speaking, non-institutionalized adults aged 25 to 74 in the 48 states—were first interviewed in 1995–1996 (M1, *n* = 7,108) and followed up in 2004–2005 (M2, *n* = 4,963, 75% of survivors). At M2, an oversample of African Americans from Milwaukee was recruited (M2 Milwaukee, *n* = 592) to increase the sample representation of African Americans. During 2011–2014, a “refresher” cohort was added to the original M1 cohort (MR, *n* = 3,577). An additional sample of Black adults from Milwaukee was added at MR (MR Milwaukee, *n* = 508). To maximize the representativeness of Blacks in the sample, we included data from the M2, M2 Milwaukee, MR, and MR Milwaukee surveys (*n* = 9,640 in total). We limit our sample to White and Black respondents who have at least one living child (*n* = 7,425).

### Measures

***Parenting type*** is a categorical variable containing three groups: (1) having children with DD, (2) having children with recent illness but no DD, and (3) having children without DD and without recent illness. Respondents were first asked whether they have children (biological, step, or adopted) and then whether each child has a developmental disability, such as autism, cerebral palsy, or epilepsy, or has had a long-term serious mental health problem. Respondents who reported having any child with any of these conditions were categorized as *having a child with DD*. Parents were categorized as *having a child with recent illness* when they did not have a child with DD but reported that their children had one of the following health problems in the past 12 months: chronic disease, disability, emotional problems, and alcohol or other substance problems. Parents who reported that their children had none of the above health problems were defined as *having a child without DD and without recent illness*.

Following prior studies (e.g., Seltzer et al., 2011; Namkung et al., 2018; Song et al., 2018), we included two ***physical health outcomes***: chronic illness and functional limitations. *Chronic illness* was measured as the total number of conditions (range: 0–27) that the respondent experienced in the past 12 months among 27 chronic and acute physical health symptoms (e.g., arthritis, diabetes, stomach problems, stroke, autoimmune disorders). *Functional limitations of daily living* was assessed as the degree of difficulty in performing nine activities of daily living (e.g., “lifting or carrying groceries,” “bathing or dressing yourself”). The Cronbach’s alpha of the nine items ranges from 0.90 to 0.92 across MIDUS samples. Originally, responses ranged from 1 (a lot) to 4 (not at all) for each item. Around 59% of individuals did not have any functional limitations and the percentages for “some” or “a lot” were small. Thus, we created a binary variable where 1 represents “some” or “a lot” of difficulty for any of the 9 items and 0 otherwise.

***Mental health*** was assessed by *Depression*. Participants were asked a series of questions regarding symptoms of depressed affect or anhedonia over a period of two or more weeks in the past year. Based on the information from the phone interview, depression diagnosis was defined according to criteria specified in the American Psychiatric Association’s Diagnostic and Statistical Manual of Mental Disorders [DSM-III-R; American Psychiatric Association (APA), 1987]. A diagnosis of Major Depression requires a period of at least 2 weeks of either depressed mood or anhedonia most of the day or nearly every day, and a series of at least four other symptoms typically found to accompany depression, including loss of interest in most activities, fatigue or loss of energy, increased or decreased appetite, insomnia, concentration problems, feelings of worthlessness and constant thoughts of death (Kessler et al., 2004). The variable was coded as 1 when a respondent felt the symptoms “every day” or “almost every day” and replied “yes” on at least five of thirteen questions (seven on depressed affect and six on anhedonia), otherwise 0. This dichotomized measurement has been referred to as the gold standard for identifying depression (Dang et al., 2020).

We included 11 ***Early-Life Adversities (ELAs)*** which may confound the association between parenting type and their health outcomes. ELAs were categorized into 3 domains: socioeconomic disadvantage, family instability, and childhood abuse. *Socioeconomic disadvantage* included three binary indicators: poorly educated parents, childhood poverty, and parental unemployment. Respondents who reported their parents’ highest education level lower than high school were coded as having poorly educated parents. Childhood poverty was measured by childhood welfare status (0 = never on welfare, 1 = ever on welfare). Respondents who had parents out of work when they wanted to be working were coded as 1 for parental unemployment, otherwise 0. *Family instability* included five binary indicators: separation from biological parents (not living with both biological parents up to age 16), parental death, sibling death, frequent moves (more than 2 times to a totally new neighborhood or town), and parental substance abuse. Those whose parents ever had problems due to the use of alcohol or drugs were defined as experiencing parental substance abuse. For *childhood abuse*, respondents were asked how often they had endured moderate physical abuse (e.g., pushed, grabbed, or shoved), severe physical abuse (e.g., kicked, bit, or hit with a fist), or emotional abuse (e.g., made insulting remarks) by parents, siblings, or anyone else. We created three indicators for each type of abuse. Responses of often or sometimes to the previous questions were coded as 1, and rarely or never were coded as 0. As less than 0.2% of respondents experienced more than 8 ELAs, we grouped these respondents together, coding ELAs as ranging from no adversity (=0) through eight or more adversities (=8).

#### Covariates

Three demographic variables were included: *age* as a continuous variable, *gender* (1 = women; 0 = men) and *race* (1 = non-Hispanic Blacks; 0 = non-Hispanic Whites). We controlled for respondent’s education level. Given the high prevalence of teen birth and high school dropout for African Americans (Winters and Winters, 2012), we dichotomized respondent’s education to establish a temporal order between this covariate and parental status (1 = less than high school diploma/GED, 0 = otherwise). *Respondent’s number of children* was derived from the question asking how many children (biological, step, or adopted) the respondent had. We also controlled for *sample* (M2 or MR), since differences in data collection across the samples and cohorts might influence the results.

Moreover, we controlled for *parental and spousal health conditions,* since individuals with multiple caregiving responsibilities may experience more compromised health than those with fewer caregiving responsibilities (Ghosh et al., 2012). Respondents were asked whether their parent had health problems in the past 12 months (1 = yes; 0 = no), including chronic disease or disability, frequent minor illness, emotional problems, and alcohol or drug problems. We aggregated all four binary variables and created a count measure of parent’s health problems (range: 0–4). *Spousal health conditions* was created by the same procedure (range: 0–4). Lastly, because bereaved parents may experience higher levels of depressive symptoms and various physical health issues, we controlled for *the experience of losing a child* (1 = yes, 0 = no).

### Analytic strategies

We first calculated descriptive statistics by race–gender group. To investigate the degree to which ELAs (each type and cumulative ELAs) are associated with parenting type (e.g., having a child with DD), we used multinomial logistic regression models. In our analysis, a child with DD includes not only a biological child but also a step or adopted child. In sensitivity analysis, we categorized children with DD into three different groups: biological (*n* = 700), adopted (*n* = 34), and stepchildren (*n* = 95). We then examined whether a similar adverse effect of ELAs was observed in these three groups in comparison to having a child without DD and recent illness. We found an increasing risk of having a DD biological child or a DD stepchild (in comparison with a child without DD or recent illness) as the number of ELAs increases (see Appendix). We also investigated whether the association between cumulative ELAs and parenting type varies by race–gender group.

To examine the association between parenting type and parental health, we employed multiple regression models with *parents with children without DD and without recent illness* as the reference category. For chronic illness, which was measured on a continuous scale, we standardized at mean of 0 and standard deviation of 1 and used ordinary least squares regression. We used logistic regression for binary outcomes, including depression and functional limitations. To investigate whether ELAs confound the association between parental status and parental health, the regression models were fitted in a stepwise approach. In Model 1, we included parenting type and all control variables (gender, race, age, education, sampling status, deceased child, spousal and parental health conditions), and we added ELAs into Model 2.

We pooled data from all respondents and first included the main effects of parenting type, gender, and race to examine whether health outcomes are significantly associated with such characteristics. Next, to test whether the association between parenting type and parental health differs across race and gender groups, we included interaction terms. More specifically, we tested (1) gender interactions to assess whether the association between parenting type and parental health varies by gender while controlling for race and other covariates, (2) race interactions to assess whether the association varies by race while controlling for gender and other covariates, and (3) finally, to investigate whether the gender–parenting type association differs by race, we conducted three-way interaction models.

The percentages of respondents having missing data for at least one of the variables of interest range from 0.1% to around 35% (e.g., parental substance abuse), due to non or partial response to the Self-Administered Questionnaire, or item-specific missingness. We followed Von Hippel’s (2009) “stratify-then-impute” method and used Stata’s *ice* command to implement 20 multiple imputations for each race–gender group separately. All analyses were implemented using Stata 16.1 (StataCorp, 2019).

## Results

### Descriptive statistics

Table 1 shows descriptive statistics by race and gender. The proportion of those having DD children is the same for Whites and Blacks—9% for men and 13% for women—indicating that women are more likely to report having DD children than men regardless of race. White women (24%) are more likely than Black women (16%) to report having recently ill children (i.e., in the past 12 months). In terms of health outcomes, compared to White parents, Black parents reported more physical health problems, but a similar risk of having depression. Women, regardless of race, reported poorer scores on all health outcomes.

**Table 1:** Means (and standard deviations) or proportions for all variables used in analysis by race and gender (*n* = 7,425).

		White (*n* = 6,110)		Black (*n* = 1,315)		Total (*n* = 7,425)
	Range	Men (*n* = 2,898)	Women (*n* = 3,212)	Men (*n* = 488)	Women (*n* = 827)	
**Parenting type**						
Child without DD^a^ or recent illness	0, 1	0.76 (*n* = 2,196)	0.63 (*n* = 2,027)	0.82 (*n* = 399)	0.72 (*n* = 591)	0.70 (*n* = 5,213)
Child with DD	0, 1	0.09 (*n* = 270)	0.13 (*n* = 411)	0.09 (*n* = 43)	0.13 (*n* = 104)	0.11 (*n* = 828)
Recently ill child	0, 1	0.15 (*n* = 432)	0.24 (*n* = 774)	0.09 (*n* = 46)	0.16 (*n* = 132)	0.19 (*n* = 1,384)
**Current health**						
Chronic illness	z-scored	−0.17	0.04	0.02	0.28	−0.01
Functional limitations	0, 1	0.33	0.44	0.42	0.54	0.41
Depression	0, 1	0.08	0.15	0.08	0.15	0.11
**Early-life adversities (ELAs)**						
Cumulative ELAs	0–8	2.04 (1.73)	2.04 (1.77)	2.61 (1.88)	2.71 (1.98)	2.18 (1.81)
*Childhood SES* ^b^						
Poorly educated parent	0, 1	0.18	0.22	0.34	0.36	0.24
Childhood poverty	0, 1	0.05	0.06	0.29	0.28	0.10
Parental unemployment	0, 1	0.15	0.14	0.15	0.12	0.14
*Childhood family instability*						
Not live with bio-parents	0, 1	0.20	0.20	0.49	0.52	0.25
Parental death	0, 1	0.08	0.09	0.16	0.15	0.10
Sibling death	0, 1	0.03	0.03	0.06	0.05	0.04
Frequent move	0, 1	0.26	0.26	0.36	0.33	0.28
Parental substance abuse	0, 1	0.19	0.22	0.21	0.21	0.21
*Childhood abuse*						
Moderate physical abuse	0, 1	0.48	0.42	0.43	0.42	0.45
Severe physical abuse	0, 1	0.24	0.17	0.30	0.32	0.22
Emotional abuse	0, 1	0.59	0.56	0.55	0.53	0.57
**Controls**						
Age	24–82	53.76 (13.38)	54.22 (13.37)	48.69 (12.77)	48.83 (12.98)	53.92 (13.14)
Poorly educated	0, 1	0.05	0.05	0.16	0.16	0.08
Number of children	0–10	2.25 (1.61)	2.37 (1.68)	2.76 (2.32)	2.47 (1.89)	2.79 (1.54)
Parental health conditions	0–4	0.49	0.63	0.51	0.59	0.54
Spousal health conditions	0–4	0.54	0.45	0.28	0.21	0.46
Deceased child	0, 1	0.05	0.07	0.10	0.10	0.07
MIDUS Refresher	0, 1	0.41	0.38	0.50	0.47	0.39

a DD: Developmentally Disabled.

b SES: Socioeconomic Status.

In terms of other covariates, Blacks, particularly Black women, experienced more ELAs than Whites, and Blacks in general were more likely to experience most domains of ELAs than Whites. Compared to Whites, the Blacks in our sample are younger, are less likely to have a high school degree or higher, and have more children. While Blacks are more likely to experience loss of a child, Whites report a higher (more adverse) score in terms of spouse’s recent health conditions.

### ELAs and parenting type

Table 2 displays the results from the multinomial logistic regression to investigate the association between ELAs and the risk of having DD children (vs. children without DD and recent illness). Model 1 presents the unique association of each ELA and the risk of having DD children. Exposure to poverty (relative risk ratio (RRR) = 1.40, 95% CI = 1.08–1.83), parental substance abuse (RRR = 1.33, 95% CI = 1.07–1.66), and emotional abuse (RRR = 1.38, 95% CI = 1.11–1.71) during childhood are associated with increased the risk of having children with DD compared to having children without DD and recent illnesses. Compared with individuals who have children without DD and recent illnesses, those whose child was recently ill are more likely to experience childhood poverty (RRR = 1.30, 95% CI = 1.02–1.65), frequent moving (RRR = 1.16, 95% CI = 1.01–1.34), severe physical abuse (RRR = 1.21, 95% CI = 1.01–1.45), and emotional abuse (RRR = 1.30, 95% CI = 1.10–1.55), but less likely to have poorly educated parents (RRR = 0.81, 95% CI = 0.69–0.95).

**Table 2:** Multinomial logistic regression (relative risk ratios, 95% ci) predicting parenting type in midlife (*n* = 7,425).

	Model 1^a^		Model 2^a^	
	DD^b^ child	Recently ill child	DD^b^ child	Recently ill child
**Demographic characteristics**				
Black	0.74^**^	0.66^***^	0.73^**^	0.61^***^
	(0.59–0.92)	(0.54–0.79)	(0.60–0.89)	(0.51–0.73)
Age	1.02^***^	1.03^***^	1.01^***^	1.03^***^
	(1.01–1.02)	(1.03–1.04)	(1.01–1.02)	(1.02–1.03)
Women	1.70^***^	2.02^***^	1.70^***^	1.98^***^
	(1.46–1.99)	(1.78–2.30)	(1.46–1.98)	(1.75–2.25)
Refresher	1.53^***^	0.94	1.51^***^	0.94
	(1.31–1.78)	(0.82–1.07)	(1.30–1.76)	(0.83–1.07)
			1.21^***^	1.11^***^
**Cumulative ELAs** ^c^			(1.16–1.26)	(1.07–1.15)
*Childhood SES*				
Parents poorly educated^d^	1.16	0.81^**^		
	(0.96–1.39)	(0.69–0.95)		
Childhood poverty	1.40^*^	1.30^*^		
	(1.08–1.83)	(1.02–1.65)		
Parental unemployment	1.06	1.13		
	(0.82–1.37)	(0.91–1.40)		
*Childhood family instability*				
Not live with bio-parents	1.18^+^	0.98		
	(0.97–1.43)	(0.83–1.16)		
Parental death	0.96	0.95		
	(0.73–1.28)	(0.76–1.19)		
Sibling Death	0.96	1.02		
	(0.62–1.50)	(0.74–1.42)		
Frequent move	1.13	1.16^*^		
	(0.93–1.36)	(1.01–1.34)		
Parental substance use	1.33^**^	1.15		
	(1.07–1.66)	(0.96–1.36)		
*Childhood abuse*				
Physical abuse	1.16	1.01		
	(0.92–1.47)	(0.84–1.22)		
Severe physical abuse	1.22^+^	1.21^*^		
	(0.97–1.54)	(1.01–1.45)		
Emotional abuse	1.38^**^	1.30^**^		
	(1.11–1.71)	(1.10–1.55)		

*** *p < 0.001;*

** *p < 0.01;*

* *p < 0.05;*

+ *p < 0.1.*

a Children without DD and recently ill is the reference category.

b DD: Developmentally Disabled.

c ELAs: Early-Life Adversities.

d Poorly educated refers to lower than high school.

**Figure 1:**
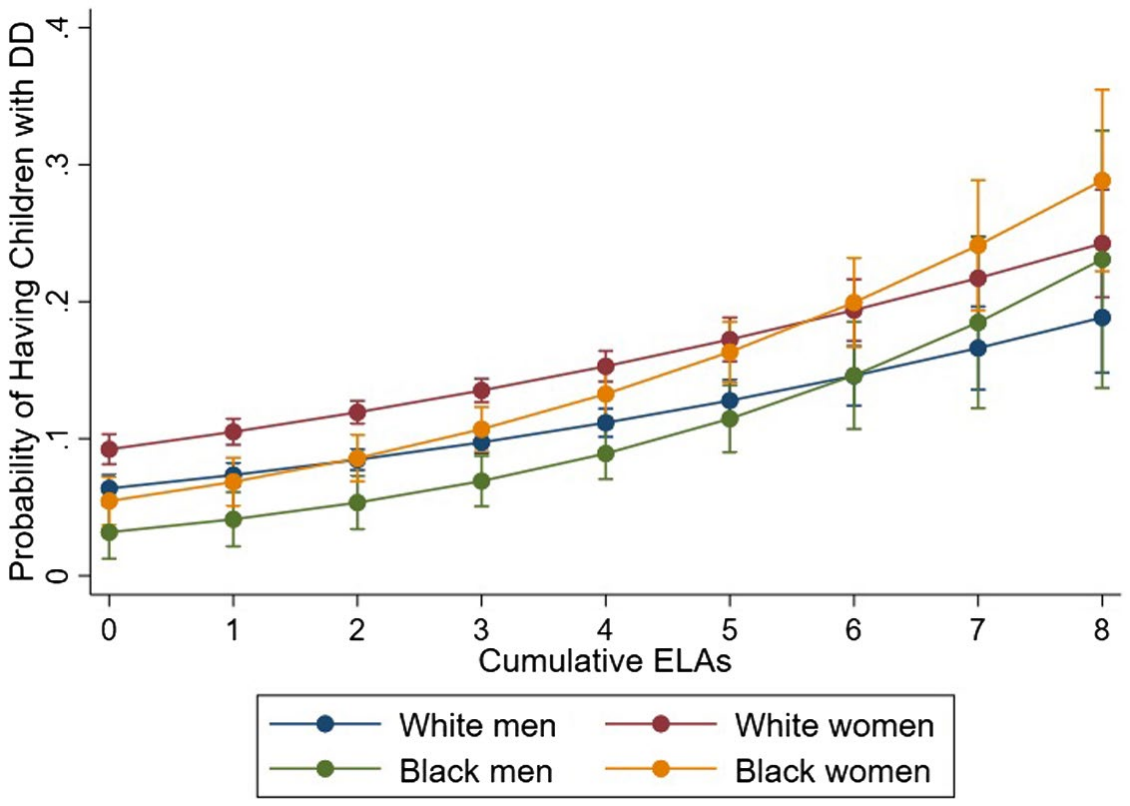
Cumulative Early-Life Adversities (ELAs) and Probability of having Children with DD. Figure is based on two logistic regressions which regressed parenting type [DD: Developmentally Disabled (DD) vs. Non-DD] on the interaction between the cumulative ELAs and race–gender groups, controlling for age and sample.

**Figure 2:**
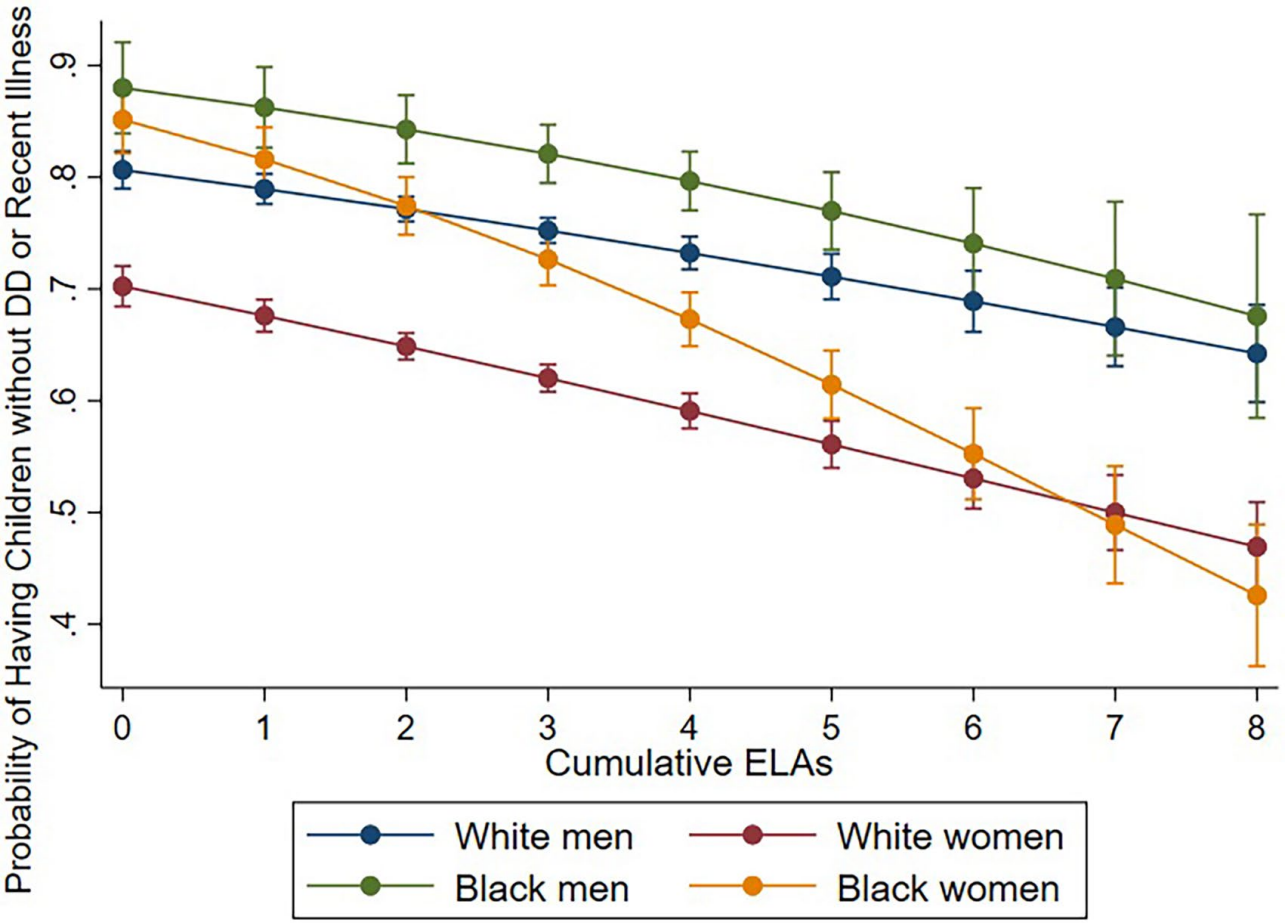
Cumulative Early-Life Adversities (ELAs) and Probability of having Children without DD and without Recent Illness. Figure is based on two logistic regressions which regressed parenting type [children without Developmentally Disabled (DD) and with recent chronic illness vs. the rest] on the interaction between the cumulative ELAs and race–gender groups, controlling for age and sample.

Model 2 shows that cumulative ELAs are significantly associated with increased risk of having children with DD (RRR = 1.21, 95% CI = 1.16–1.26), and children who were recently ill (RRR = 1.11, 95% CI = 1.07–1.15), compared to children without DD and recent illness. To examine heterogeneous effects across race–gender groups, we fit two logistic regression models by regressing parenting type (DD vs. Non-DD; Children without DD and without recent illness vs. the other types) on the interaction between the cumulative ELA index and race–gender groups. As the number of ELAs increases, the probability of having children *with* DD increases (Figure 1) while the probability of having children *without* health issues (DD or recent illness) decreases for all race–gender groups (Figure 2). Notably, the steeper slope for Black women in Figure 2 indicates that the negative association between having ELAs and having children without DD and recent illness is stronger among Black women, compared to White women and White men (*p* < 0.05). No significant difference was found between Black women and Black men.

### Race and gender differences in associations between parenting types and health outcomes

Tables 3-5 present the associations between parental type and health outcomes of the entire sample. Model 1 includes all covariates except for ELAs while Model 2 includes ELAs to test whether ELAs are a potential confounder. We started with testing the main effects of parenting type followed by gender interaction, race interaction, and three-way interaction effects.

**Table 3:** Estimates of the effects of parenting type and interactions on chronic illness (*n* = 7,425)^a^.

	Parenting type effect		Gender interaction		Race interaction		Three-way interactions	
	Model 1	Model 2	Model 1	Model 2	Model 1	Model 2	Model 1	Model 2
DD^b^	0.30^***^	0.27^***^	0.16^*^	0.13^*^	0.17	0.18	0.19^**^	0.16^*^
	(0.22–0.38)	(0.19–0.35)	(0.04–0.28)	(0.01–0.25)	(−0.07–0.42)	(−0.07–0.42)	(0.06–0.32)	(0.04–0.29)
Sick	0.27^***^	0.24^***^	0.19^***^	0.16^***^	0.04	0.03	0.16^**^	0.12^*^
	(0.22–0.33)	(0.18–0.29)	(0.10–0.28)	(0.07–0.25)	(−0.15–0.23)	(−0.16–0.21)	(0.06–0.25)	(0.03–0.22)
Women	0.23^***^	0.23^***^	0.18^***^	0.18^***^	0.23^***^	0.23^***^	0.17^***^	0.18^***^
	(0.18–0.27)	(0.18–0.27)	(0.12–0.23)	(0.13–0.24)	(0.18–0.27)	(0.18–0.27)	(0.11–0.24)	(0.12–0.24)
Black	0.29^***^	0.24^***^	0.29^***^	0.25^***^	0.25^***^	0.21^***^	0.24^***^	0.20^***^
	(0.22–0.35)	(0.18–0.31)	(0.23–0.35)	(0.18–0.31)	(0.18–0.32)	(0.14–0.28)	(0.13–0.34)	(0.10–0.31)
DD ^*^ Women			0.24^**^	0.24^**^			0.16^*^	0.16+
			(0.10–0.39)	(0.09–0.38)			(0.00–0.32)	(−0.00–0.32)
Sick ^*^ Women			0.14^*^	0.13^*^			0.15^*^	0.15^*^
			(0.02–0.25)	(0.02–0.24)			(0.03–0.27)	(0.03–0.27)
DD ^*^ Black					0.11	0.08	−0.25	−0.27
					(−0.08–0.30)	(−0.11–0.27)	(−0.59–0.08)	(−0.60–0.06)
Sick ^*^ Black					0.20^*^	0.19^*^	0.32^*^	0.33^*^
					(0.05–0.36)	(0.03–0.34)	(0.03–0.61)	(0.05–0.62)
Women ^*^ Black							0.02 (−0.11–0.16)	0.03 (−0.11–0.16)
DD ^*^ Women ^*^ Black							0.49^*^ (0.10–0.88)	0.47^*^ (0.09–0.86)
Sick ^*^ Women ^*^ Black							−0.18 (−0.53–0.16)	−0.23 (−0.57–0.12)
ELAs^c^ index		0.07^***^		0.07^***^		0.07^***^		0.07^***^
		(0.06–0.08)		(0.06–0.08)		(0.06–0.08)		(0.06–0.08)

*** *p* < 0.001;

** *p* < 0.01;

* *p* < 0.05;

+ *p* < 0.1.

a Ordinary Least Square regression model. Standardized coefficients and the 95% confidence interval (in parentheses) are reported.

b DD: Developmentally Disabled.

c ELAs: Early-Life Adversities.Model 1 and Model 2 include sociodemographic controls.

In the first set of models (Parenting type effect in Table 3), parents with DD children (*β* = 0.30; *p* < 0.001) as well as recently ill children (*β* = 0.27; *p* < 0.001) have higher levels of chronic illnesses than parents with children without health issues (Model 1). After controlling for ELAs, the associations remain significant while the effect sizes decrease moderately (Model 2). Next, the models with a gender interaction show that the positive association between having DD children and the number of chronic illnesses is significantly stronger for women than men even after controlling for ELAs (*β* = 0.24; *p* < 0.01). Additionally, the gap between chronic illnesses of parents with recently ill children and parents with children without health issues is stronger for women than men (*β* = 0.14 in Model 1; *p* < 0.05, *β* = 0.13 in Model 2; *p* < 0.05).

There is no significant racial difference in the gap of chronic illnesses between parents with DD children and parents with children without health issues, yet the positive association between having recently ill children and the number of chronic illnesses is significantly stronger for Blacks than Whites (*β* = 0.20 in Model 1; *p* < 0.05, *β* = 0.19 in Model 2; *p* < 0.05). Lastly, results from three-way interactions show that an interaction between gender (Women vs. Men) and parenting type (having DD children vs. children without health issues) is stronger for Blacks than Whites (*β* = 0.49 in Model 1; *p* < 0.05). The finding remains significant even after controlling for ELAs in Model 2. Such differences are not statistically significant for parents having recently ill children vs. parents with children that have no health issues.

Results are similar for functional limitations in Table 4. Parents with DD children (OR = 1.63 in Model 1; *p* < 0.001, OR = 1.50 in Model 2; *p* < 0.001) as well as parents with recently ill children (OR = 2.29 in Model 1; *p* < 0.001, OR = 2.11 in Model 2; *p* < 0.001) have a higher risk of experiencing functional limitations than parents with children without health issues even after controlling for ELAs. The gender interaction model shows that the positive association between having DD children and the risk of having functional limitations is stronger for women than men even after controlling for ELAs (OR = 1.56 in Model 2; *p* < 0.01). The gap between functional limitations of parents with recently ill children and parents with children without health issues is larger for women than men but not statistically significant as we control for ELAs.

**Table 4:** Estimates of the effects of parenting type and interactions on functional limitations (*n* = 7,425)^a^.

	Parenting type effect		Gender interaction		Race interaction		Three-way interactions	
	Model 1	Model 2	Model 1	Model 2	Model 1	Model 2	Model 1	Model 2
DD^b^	1.63^***^	1.50^***^	1.24	1.15	1.12	1.17	1.36^*^	1.27+
	(1.39–1.92)	(1.27–1.77)	(0.96–1.61)	(0.88–1.49)	(0.67–1.89)	(0.69–1.98)	(1.03–1.79)	(0.96–1.68)
Sick	2.29^***^	2.11^***^	1.99^***^	1.85^***^	4.45^***^	4.47^***^	2.09^***^	1.94^***^
	(2.01–2.61)	(1.85–2.41)	(1.61–2.45)	(1.50–2.29)	(2.86–6.94)	(2.85–7.00)	(1.67–2.60)	(1.55–2.43)
Women	1.55^***^	1.58^***^	1.41^***^	1.44^***^	1.55^***^	1.58^***^	1.39^***^	1.42^***^
	(1.40–1.72)	(1.42–1.75)	(1.25–1.60)	(1.27–1.63)	(1.40–1.72)	(1.42–1.75)	(1.21–1.59)	(1.24–1.63)
Black	2.12^***^	1.92^***^	2.13^***^	1.93^***^	2.23^***^	2.05^***^	2.18^***^	2.01^***^
	(1.85–2.44)	(1.67–2.20)	(1.86–2.45)	(1.67–2.21)	(1.91–2.60)	(1.75–2.40)	(1.72–2.77)	(1.59–2.56)
DD ^*^ Women			1.57^**^	1.56^**^			1.26	1.26
			(1.13–2.18)	(1.12–2.18)			(0.88–1.81)	(0.88–1.82)
Sick ^*^ Women			1.27+	1.25			1.33^*^	1.33+
			(0.97–1.65)	(0.95–1.63)			(1.00–1.77)	(1.00–1.77)
DD ^*^ Black					1.38	1.24	0.54	0.49^+^
					(0.90–2.10)	(0.81–1.91)	(0.26–1.13)	(0.23–1.04)
Sick ^*^ Black					0.55^**^	0.51^***^	0.60	0.60
					(0.38–0.80)	(0.35–0.75)	(0.30–1.18)	(0.30–1.20)
Women ^*^ Black							1.06 (0.78–1.43)	1.05 (0.78–1.43)
DD ^*^ Women ^*^ Black							3.98^**^ (1.57–10.07)	3.90^**^ (1.53–9.93)
Sick ^*^ Women ^*^ Black							0.86 (0.38–1.96)	0.77 (0.34–1.78)
ELAs^c^ index		1.22^***^		1.22^***^		1.22^***^		1.22^***^
		(1.18–1.25)		(1.18–1.25)		(1.18–1.26)		(1.18–1.26)

*** *p* < 0.001;

** *p* < 0.01;

* *p* < 0.05;

+ *p* < 0.1

a Logistic regression model. Odds ratio and the 95% confidence interval (in parentheses) are reported.

b DD: Developmentally Disabled.

c ELAs: Early-Life Adversities. Model 1 and Model 2 include sociodemographic controls.

No significant racial difference in functional limitations between parents with DD children and parents with children without health issues was found in the model with a race interaction; however, the positive association between having recently ill children and the number of functional limitations is significantly stronger for Blacks than Whites (OR = 0.55 in Model 1; *p* < 0.01, OR = 0.51 in Model 2; *p* < 0.001). Lastly, results from three-way interactions show an interaction between gender (Women vs. Men) and parenting type (having DD children vs. children without health issues) is stronger for Blacks than Whites, which is statistically significant even after controlling for ELAs (OR = 3.90 in Model 2; *p* < 0.01). A three-way interaction term was not statistically significant as we investigate the effect of having recently ill children vs. parents with children that have no health issues.

For depression (in Table 5), both parents with DD children (OR = 2.25 in Model 1; *p* < 0.001, OR = 2.06 in Model 2; *p* < 0.001) and parents with recently ill children (OR = 1.78 in Model 1; *p* < 0.001, OR = 1.60 in Model 2; *p* < 0.001) have higher levels of depression than parents with children without health issues even after controlling for ELAs. Yet, neither gender nor racial differences in the effect of having DD or ill children are statistically significant. Further, there is no significant three-way interaction effect.

**Table 5:** Estimates of the effects of parenting type and interactions on depression (*n* = 7,425)^a^.

	Parenting type effect		Gender effect		Race effect		Three-way interactions	
	Model 1	Model 2	Model 1	Model 2	Model 1	Model 2	Model 1	Model 2
DD^b^	2.25^***^	2.06^***^	2.24^***^	2.10^***^	2.56^**^	2.64^**^	2.41^***^	2.28^***^
	(1.82–2.77)	(1.66–2.55)	(1.53–3.26)	(1.44–3.07)	(1.33–4.95)	(1.36–5.15)	(1.62–3.59)	(1.53–3.40)
Sick	1.78^***^	1.60^***^	1.77^**^	1.63^**^	1.72^+^	1.67	1.83^**^	1.69^**^
	(1.48–2.15)	(1.32–1.94)	(1.23–2.53)	(1.13–2.34)	(0.93–3.17)	(0.89–3.10)	(1.26–2.67)	(1.16–2.47)
Women	2.06^***^	2.08^***^	2.05^***^	2.10^***^	2.06^***^	2.08^***^	2.07^***^	2.11^***^
	(1.74–2.43)	(1.76–2.45)	(1.67–2.52)	(1.70–2.58)	(1.74–2.43)	(1.76–2.45)	(1.64–2.60)	(1.68–2.65)
Black	0.86	0.75^**^	0.86	0.75^**^	0.87	0.78^+^	0.90	0.81
	(0.70–1.05)	(0.61–0.93)	(0.70–1.05)	(0.61–0.93)	(0.67–1.12)	(0.61–1.01)	(0.57–1.41)	(0.52–1.28)
DD ^*^ Women			1.01	0.97			0.93	0.91
			(0.64–1.57)	(0.62–1.52)			(0.57–1.50)	(0.56–1.48)
Sick ^*^ Women			1.01	0.98			0.96	0.94
			(0.67–1.54)	(0.65–1.49)			(0.62–1.49)	(0.60–1.46)
DD ^*^ Black					0.90	0.81	0.55	0.52
					(0.53–1.52)	(0.47–1.38)	(0.17–1.80)	(0.16–1.72)
Sick ^*^ Black					1.04	0.97	0.67	0.68
					(0.62–1.72)	(0.580–1.621)	(0.18–2.48)	(0.18–2.50)
Women ^*^ Black							0.95 (0.56–1.62)	0.95 (0.55–1.62)
DD ^*^ Women ^*^ Black							1.87 (0.49–7.07)	1.76 (0.46–6.73)
Sick ^*^ Women ^*^ Black							1.68 (0.41–6.92)	1.55 (0.37–6.46)
ELAs^c^ index		1.20^***^		1.20^***^		1.20^***^		1.20^***^
		(1.15–1.24)		(1.15–1.24)		(1.15–1.25)		(1.15–1.24)

*** *p* < 0.001;

** *p* < 0.01;

* *p* < 0.05;

+ *p* < 0.1

a Logistic regression model. Odds ratio and the 95% confidence interval (in parentheses) are reported.

b DD: Developmentally Disabled.

c ELAs: Early-Life Adversities. Model 1 and Model 2 include sociodemographic controls.

**Figure 3:**
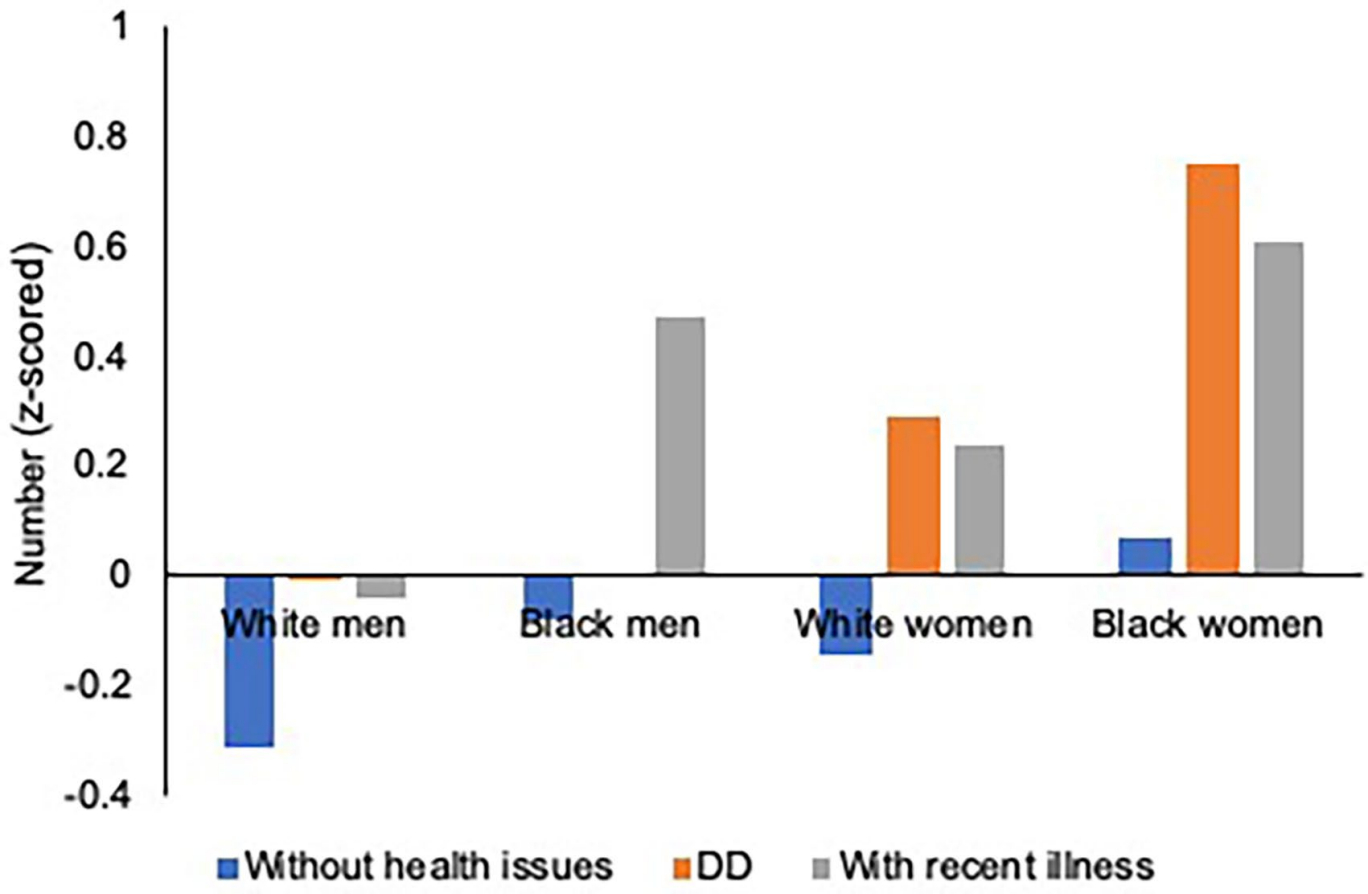
Parental Health by Race, Gender, and Parenting Type: Chronic Illnesses. Figure is based on the three-way interaction results in Table 3. Controlled for covariates and cumulative early life adversities (ELAs). DD refers to DD: Developmentally Disabled.

**Figure 4:**
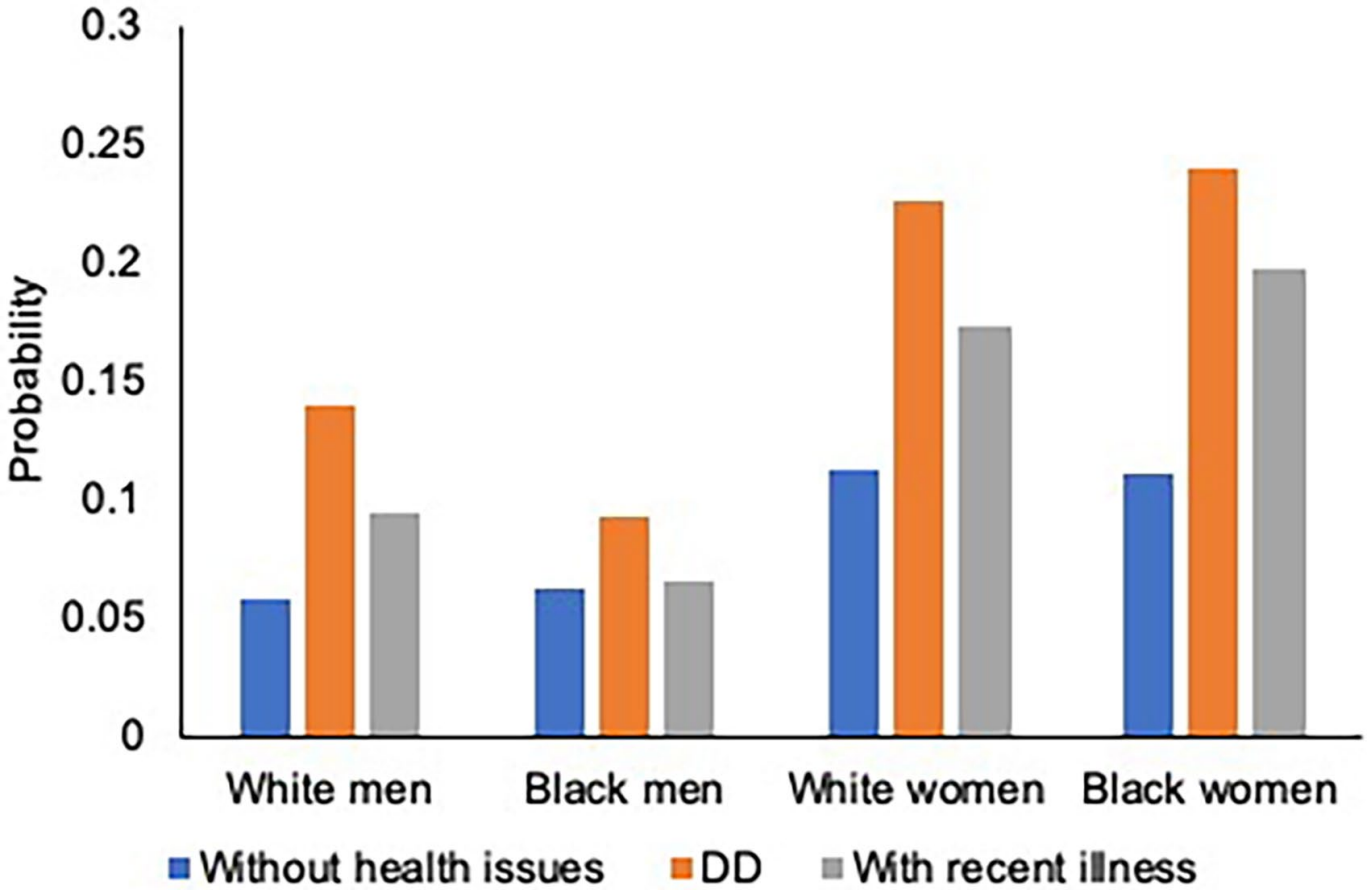
Parental Health by Race, Gender, and Parenting Type: Functional Limitations. Figure was based on the three-way interaction results in Table 4. Controlled for covariates and cumulative early life adversities (ELAs). DD refers to DD: Developmentally Disabled.

**Figure 5:**
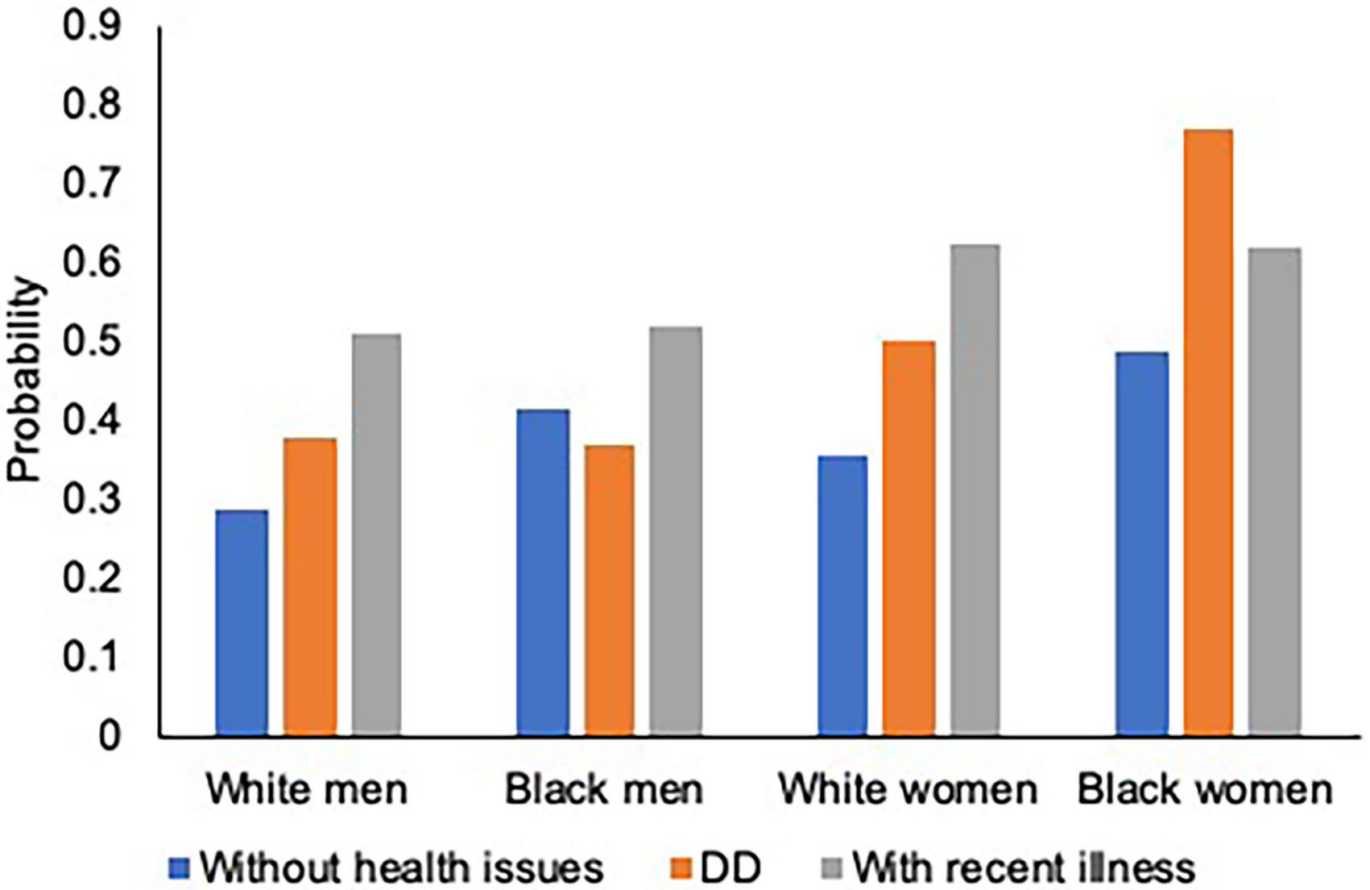
Parental Health by Race, Gender, and Parenting Type: Depression. Figure was based on the three-way interaction results in Table 5. Controlled for covariates and cumulative early life adversities (ELAs). DD refers to DD: Developmentally Disabled.

Figures 3–5 illustrate race–gender nuances in our findings. As for physical health outcomes (number of chronic illnesses and probability of having a functional limitation), the gap between parents with DD children and those with children without health issues is stronger for women than men. The finding is consistent across racial groups, suggesting that having DD children burdens women more heavily than men, regardless of race. Moreover, such a gender disparity was stronger for Blacks than Whites, suggesting that Black women’s physical health is more adversely affected than other race–gender groups by having DD children. As for the mental health outcome, regardless of gender and race, parents with DD children show a higher risk of having depression than those with children without health issues.

## Discussion

This study yields several key findings. First, the current study is among the few studies that explain the extent to which the parents’ early-life experiences are associated with the risk of having DD children and the health of parents. Our findings suggest that exposure to cumulative ELAs and individual ELAs (childhood poverty, parental substance abuse, and emotional abuse) are associated with an elevated risk of having a DD child. The association between cumulative ELAs and having a DD child are robust with different operationalizations of parenting types. That is, the risk of having a biological DD child or a step DD child may increase for those who experienced ELAs. This echoes previous research on how parents’ adverse childhood experiences compound over time and are negatively associated with their physical and mental health throughout the life course (Nelson et al., 2020) as well as risks for offspring’s development (Bowers and Yehuda, 2016; McDonnell and Valentino, 2016; Sun et al., 2017; Folger et al., 2018). Individuals who experienced ELAs might also have a greater chance of having a DD stepchild, as similarities in social, interpersonal, and cultural backgrounds between individuals are underlying components of partner selection (Merikangas, 1982; McLeod, 1995; Lindeman et al., 2002; Butterworth and Rodgers, 2008). This result deepens our understanding of the theme of “linked lives,” which emphasizes the interdependence of individuals across generations within a family (Elder, 1994). (Dis)advantages rooted in early-life experiences can accumulate throughout the life course (Ferraro and Shippee, 2009), and parents’ exposure to childhood adversities may even jeopardize their children’s health.

Moreover, our findings show that ELAs are disproportionally associated with the risk of having unhealthy children across race–gender groups. That is, with more adversities, the probability of having unhealthy children (i.e., either DD or recent illness) increases for all parents, but most steeply for Black women. Our findings support previous conceptual and empirical work on how exposure to negative and distressed environments disproportionately affects Black women (Geronimus et al., 2006). Black women, possessing multiple subordinate-group identities, have a higher chance of being exposed to varying social, familial, and economic adversities throughout the life course (Pager and Shepherd, 2008; Purdie-Vaughns and Eibach, 2008; Brown et al., 2016), which might place them at a greater risk of having and caring for children with health issues.

Consistent with prior work on caregiving children and health of the parents (e.g., Pinquart and Sörensen, 2006; Dunn et al., 2019), we found that having DD children is associated with elevated risks of compromised physical and mental health of parents. Yet, the association varies by gender and race of parents, and gender and racial disparities differ across health outcomes. While having DD children has adverse effects on physical health more for women than men for both races, no such gendered pattern was observed on mental health. Although fathers have in recent years become more involved with their children while also fulfilling their traditional role as a financial provider (Parker and Wang, 2013), mothers still have more household responsibilities, including chores and caregiving, and are sometimes burdened by becoming a financial provider as well (e.g., Blair-Loy et al., 2015). Maintaining their traditional mother role and sometimes participating as an economic provider might have compromised the health of women with children with DD.

In terms of mental health, we found that parents with children having DD as well as recent illness have higher levels of depression than parents with children without health issues. Yet, there were no gender differences. That is, in contrast to our findings in physical health, women with DD children do not report worse mental health than their male counterparts. The stress process model provides a framework for understanding not only the social origins of parents’ stress, but also how stressors as well as strains are shaped by parents’ social status and roles in society (Pearlin, 1989). In addition, the stress process model takes into account the role of coping resources (Pearlin, 1989). For example, having high levels of psychosocial resources such as social support (Turner and Jay Turner, 2013) and self-esteem (Werner and Shulman, 2013) yields significant improvements in mental health as well as quality of life. These psychosocial resources may enable individuals who confront stress-provoking conditions to better manage these difficult situations.

Indeed, recent work has shown that parenting DD children has positive aspects (Beighton and Wills, 2017), which might differ by gender. Mothers may experience more positive experiences from caring for DD children than fathers, which is consistent with a previous study showing higher levels of caregiving satisfaction by mothers than fathers (Pruchno and Patrick, 1999). Having social resources, such as informal social support and community resources, may help women to alleviate the mental health burden of caregiving for DD children. For Blacks, despite frequent exposure to racial discrimination, they may have developed better coping resources to offset the adversity (Louie et al., 2022). However, there are few empirical studies that investigate the role of these psychosocial coping resources. Future studies need to identify underlying mechanisms that alleviate the mental health burdens of parents (particularly women and women of color) with a DD child.

Consistent with prior work (Magaña and Smith, 2006; Ha et al., 2011), we found that Black women with DD children suffer from more chronic illnesses and a greater risk of having functional limitations than other race–gender groups. This suggests that Black women might have a higher caregiving burden than other race–gender groups, with fewer material resources to cope with the stresses of having DD children. That is, Black women, who are doubly marginalized (Purdie-Vaughns and Eibach, 2008), may have particularly high exposures to discrimination, economic deprivation (Pager and Shepherd, 2008), and other life adversities (e.g., Bowleg, 2012), thus resulting in the worst health profiles across race–gender groups (Geronimus et al., 2006). Such structural risks and inequalities have led Black mothers to be exposed to risks and disadvantages thereby suffering from caregiving burden and negative health outcomes in later life.

Finally, we found that the mental and physical health burdens of having a DD child are relatively smaller for Black men compared to the other race–gender groups. There might be two reasons for this. First, understanding the impact of having children with DD for Black men can be more complicated due to persistent negative stereotypes, such as their being non-residential and non-custodial (Chetty et al., 2019). Black men are more likely to experience financial hardships from job loss and difficulties in providing social and economic resources for their family than White men. These ecological and social factors might contribute toward Black men being non-resident fathers, being less involved with caring for a DD child and, as a result, experiencing fewer health problems than Black women and Whites. Importantly, we also cannot rule out lack of statistical power to detect the impact of having a DD child for Black fathers since only 6.5% of our sample is Black men (compared to 11% Black women), with only 43 Black fathers having a DD child.

## Limitations

Our research has several limitations to be acknowledged. First, the symptoms and limitations of DD children differ based on their type of disability. Due to a small sample size of each type of DD, this study did not investigate the effect of each type of DD on parents’ health. Future research should include a more refined measure that would allow the effects of various disabilities to be examined. Second, the majority of the sample of Black parents was derived from the Milwaukee MIDUS data. Therefore, the sample is not representative of Black parents in the U.S. Nonetheless, the Milwaukee sample offers an informative context for understanding racial disparities in health, as many Black adults live in segregated urban contexts. Third, other possible unobserved confounders limit the interpretation of our findings. For example, genetics and biological deficit are salient factors, yet we were not able to include potential genetic influences or biomarkers that are associated with the risk of having a DD child and the health of parents. Lastly, despite a clear temporal order to our variables in theory, we did not attempt to draw any causal inferences, as these variables (e.g., ELAs) were retrospectively measured and reported in midlife, and imperfect retrospective recall may have biased respondents’ reports.

Despite such limitations, this study is among only a few that have applied the intersectionality approach and life-course perspective to the study of caregiving and health disparities using a sample of Black and White parents of DD children. We advance prior work by adding the role of parental history of ELAs to better understand its impact on the risk of having DD children and later parental health outcomes across racial and gender groups. Policy programs initiated in early life to minimize exposure to poor environments and multiple adversities may enhance the health of disadvantaged children as well as their offspring. More importantly, policy programs that mitigate the burden on parents and caregivers of children with DD throughout the life course are critical. Our results show that while raising children with DD is difficult and burdensome for most parents, the burden might be larger for Black mothers as they are more likely to be situated in a multiply marginalized position, with greater adversities and fewer resources throughout the life course. Future policy programs should provide targeted interventions for minority parents who care for special needs children.

## Author’s note

A draft form of presentation titled “Child’s Developmental Disabilities and Parental Health in Later Life: Do parental Race and Gender Matter?” was presented at the Gerontological Society of America (GSA) (online).

## Data availability statement

Publicly available datasets were analyzed in this study. The datasets analyzed for this study can be found in the Midlife in the United States study at: http://www.midus.wisc.edu. The datasets generated for this study are available on request to the corresponding author.

## Ethics statement

The studies involving human participants were reviewed and approved by Education, Social/Behavioral Sciences, and Health Institutional Review Boards at the University of Wisconsin-Madison. The patients/participants provided their written informed consent to participate in this study.

## Author contributions

JL: conceptualization, methodology, data curation, investigation, validation, visualization, and writing—original draft preparation. MG: methodology, data curation, investigation, validation, visualization, and writing—reviewing and editing. CL: conceptualization, methodology, validation, visualization, writing—reviewing and editing, funding acquisition, project administration, resources, and supervision. All authors contributed to the article and approved the submitted version.

## Funding

This work was supported by the National Institutes of Health (grant number R00AG052458).

## Conflict of interest

The authors declare that the research was conducted in the absence of any commercial or financial relationships that could be construed as a potential conflict of interest.

## Publisher’s note

All claims expressed in this article are solely those of the authors and do not necessarily represent those of their affiliated organizations, or those of the publisher, the editors and the reviewers. Any product that may be evaluated in this article, or claim that may be made by its manufacturer, is not guaranteed or endorsed by the publisher.
