# Supplementary material for: Gendered racial disparities in health of parents with children with developmental disabilities
Source: Front Psychol. 2022 Sep 2;13:926655. doi: 10.3389/fpsyg.2022.926655 (PMC9479760; doi:10.3389/fpsyg.2022.926655)
Supplement: Supplementary file 1 [file Table_1.docx]

**Appendix**

Table A1. Characteristics of Developmentally Disabled Children (Mean and Standard Deviation; Proportion and Sample size) by Race and Gender of their Parents^a^ (n=696)

|  |  | Range | Whites | | Blacks | | Total |
| --- | --- | --- | --- | --- | --- | --- | --- |
|  |  |  | Men | Women | Men | Women |  |
| Gender | Boys | 0, 1 | 0.53  (n=124) | 0.53  (n=181) | 0.48  (n=18) | 0.51  (n=40) | 0.52  (n=363) |
|  | Girls | 0, 1 | 0.47  (n=111) | 0.47  (n=163) | 0.51  (n=19) | 0.48  (n=38) | 0.48  (n=331) |
|  |  |  |  |  |  |  |  |
| Relations to the parents^b^ | Biological | 0, 1 | 0.78  (n=183) | 0.86  (n=292) | 0.81  (n=30) | 0.97  (n=76) | 0.84  (n=587) |
|  | Adopted | 0, 1 | 0.06  (n=15) | 0.04  (n=12) | 0.03  (n=1) | 0.00  (n=0) | 0.04  (n=29) |
|  | Step | 0, 1 | 0.15  (n=36) | 0.11  (n=37) | 0.16  (n=6) | 0.03  (n=2) | 0.12  (n=81) |
|  |  |  |  |  |  |  |  |
| Residential Status | Live with the parents | 0, 1 | 0.30  (n=70) | 0.32  (n=109) | 0.19  (n=7) | 0.38  (n=30) | 0.31  (n=216) |
|  | Do not live with the parents | 0, 1 | 0.70  (n=165) | 0.68  (n=235) | 0.81  (n=30) | 0.62  (n=49) | 0.69  (n=479) |
|  | |  |  |  |  |  |  |
| Age^c^ | | 0-63 | 29.82  (13.41)  (n=222) | 32.02  (14.32)  (n=335) | 29.14  (14.60)  (n=36) | 28.72  (13.38)  (n=79) | 30.75  (13.97)  (n=672) |

Notes: a. n=696 after limiting the sample to parents with a single DD child.

b. Respondents who identified the relationship to the DD child as “foster” and “other child” were not included (n=4)

c. In MIDUS Refresher Milwaukee, 15th child age is missing (missing = 25). Also, considering the age of the parents with DD children, we limited the child’s age to 63 years old while the oldest age of the child reported was 120. The total number of respondents for the Age variable is 679.

Table A2. Multinomial Logistic Regression (Relative Risk Ratios, 95% CI) Predicting Parenting Type in Midlife (n=7,417)^a^

|  | Model 1 | | | | Model 2 | | | |
| --- | --- | --- | --- | --- | --- | --- | --- | --- |
|  | Recently  ill Child  (n=1,384) | Biological DD^b^ Child  (n=700) | Adopted  DD Child  (n=34) | Step  DD Child  (n=95) | Recently  ill Child (n=1,384) | Biological DD Child  (n=700) | Adopted  DD Child (n=34) | Step  DD Child  (n=95) |
| **Demographic Characteristics** |  |  |  |  |  |  |  |  |
| Black | 0.66*** | 0.82 | 0.16+ | 0.38* | 0.61*** | 0.81+ | 0.14+ | 0.38** |
|  | (0.54 – 0.80) | (0.65 – 1.04) | (0.02 – 1.24) | (0.18 – 0.79) | (0.51 - 0.73) | (0.66 - 1.00) | (0.02 - 1.05) | (0.19 - 0.75) |
| Age | 1.03*** | 1.02*** | 1.02 | 0.99 | 1.03*** | 1.02*** | 1.02+ | 0.99 |
|  | (1.02 – 1.04) | (1.01 – 1.03) | (0.99 – 1.05) | (0.97 – 1.01) | (1.02 - 1.03) | (1.01 - 1.02) | (1.00 - 1.05) | (0.97 - 1.00) |
| Women | 2.03*** | 1.97*** | 0.81 | 0.85 | 1.98*** | 1.96*** | 0.8 | 0.85 |
|  | (1.78 – 2.31) | (1.65 – 2.34) | (0.40 – 1.65) | (0.56 – 1.29) | (1.75 - 2.25) | (1.66 - 2.32) | (0.40 - 1.61) | (0.56 - 1.29) |
| Refresher | 0.93 | 1.50*** | 1.44 | 1.77* | 0.94 | 1.49*** | 1.36 | 1.75** |
|  | (0.82 – 1.07) | (1.27 – 1.79) | (0.69 – 2.99) | (1.14 – 2.74) | (0.83 - 1.07) | (1.26 - 1.76) | (0.67 - 2.77) | (1.15 - 2.67) |
| **Cumulative ELAs^c^** |  |  |  |  | 1.11*** | 1.23*** | 1.07 | 1.19** |
|  |  |  |  |  | (1.07 - 1.15) | (1.18 - 1.29) | (0.87 - 1.32) | (1.06 - 1.32) |
| ***Childhood SES*** |  |  |  |  |  | | | |
| Parents poorly educated^d^ | 0.81** | 1.16 | 1.47 | 1.07 |  | | | |
|  | (0.70 - 0.95) | (0.95 - 1.42) | (0.65 - 3.32) | (0.62 - 1.84) |  | | | |
| Childhood poverty | 1.31* | 1.43* | 0.00 | 1.48 |  | | | |
|  | (1.03 - 1.68) | (1.08 - 1.89) | (0.000 - .) | (0.73 - 3.02) |  | | | |
| Parental unemployment | 1.12 | 1.05 | 0.99 | 1.17 |  | | | |
|  | (0.89 - 1.42) | (0.80 - 1.37) | (0.25 - 3.86) | (0.59 - 2.32) |  | | | |
| ***Childhood family instability*** |  |  |  |  |  | | | |
| Not live with bio-parents | 0.97 | 1.17 | 1.67 | 1.15 |  | | | |
|  | (0.82 - 1.15) | (0.95 - 1.45) | (0.67 - 4.19) | (0.67 - 1.98) |  | | | |
| Parental death | 0.94 | 0.94 | 1.00 | 1.24 |  | | | |
|  | (0.75 - 1.18) | (0.68 - 1.29) | (0.23 - 4.32) | (0.58 - 2.66) |  | | | |
| Sibling Death | 0.99 | 0.88 | 1.12 | 0.96 |  | | | |
|  | (0.71 - 1.38) | (0.54 - 1.44) | (0.15 - 8.66) | (0.21 - 4.28) |  | | | |
| Frequent move | 1.17* | 1.17 | 0.7 | 1.1 |  | | | |
|  | (1.01 - 1.35) | (0.96 - 1.43) | (0.27 - 1.85) | (0.66 - 1.84) |  | | | |
| Parental substance use | 1.15 | 1.39** | 0.68 | 1.33 |  | | | |
|  | (0.97 - 1.38) | (1.12 - 1.74) | (0.16 - 2.88) | (0.70 - 2.54) |  | | | |
| ***Childhood abuse*** |  |  |  |  |  | | | |
| Physical abuse | 1.00 | 1.11 | 1.23 | 1.58 |  | | | |
|  | (0.84 - 1.20) | (0.85 - 1.45) | (0.47 - 3.22) | (0.87 - 2.87) |  | | | |
| Severe physical abuse | 1.21* | 1.28* | 0.91 | 0.93 |  | | | |
|  | (1.00 - 1.45) | (1.00 - 1.64) | (0.31 - 2.69) | (0.51 - 1.69) |  | | | |
| Emotional abuse | 1.31** | 1.47** | 1.63 | 0.98 |  | | | |
|  | (1.11 - 1.55) | (1.16 - 1.88) | (0.62 - 4.32) | (0.54 - 1.78) |  | | | |

Notes: *** p<0.001, ** p<0.01, * p<0.05, + p<0.1

a. Children without DD and recent illness are the reference category (n=5,213).

b. DD: Developmentally Disabled.

c. ELAs: Early-Life Adversities.

d. Poorly educated refers to lower than high school.

Figure A1. Cumulative Early-Life Adversities (ELAs) and Probability of Having Biological Children with DD

Notes: Figure 1 is based on two logistic regressions which regressed parenting type (Biological DD: Developmentally Disabled [DD] versus Children without DD and recent illness) on the interaction between the cumulative ELAs and race–gender groups, controlling for age and sample.
