# Supplementary figures and images for: Gendered racial disparities in health of parents with children with developmental disabilities
Source: Front Psychol. 2022 Sep 2;13:926655. doi: 10.3389/fpsyg.2022.926655 (PMC9479760; doi:10.3389/fpsyg.2022.926655)

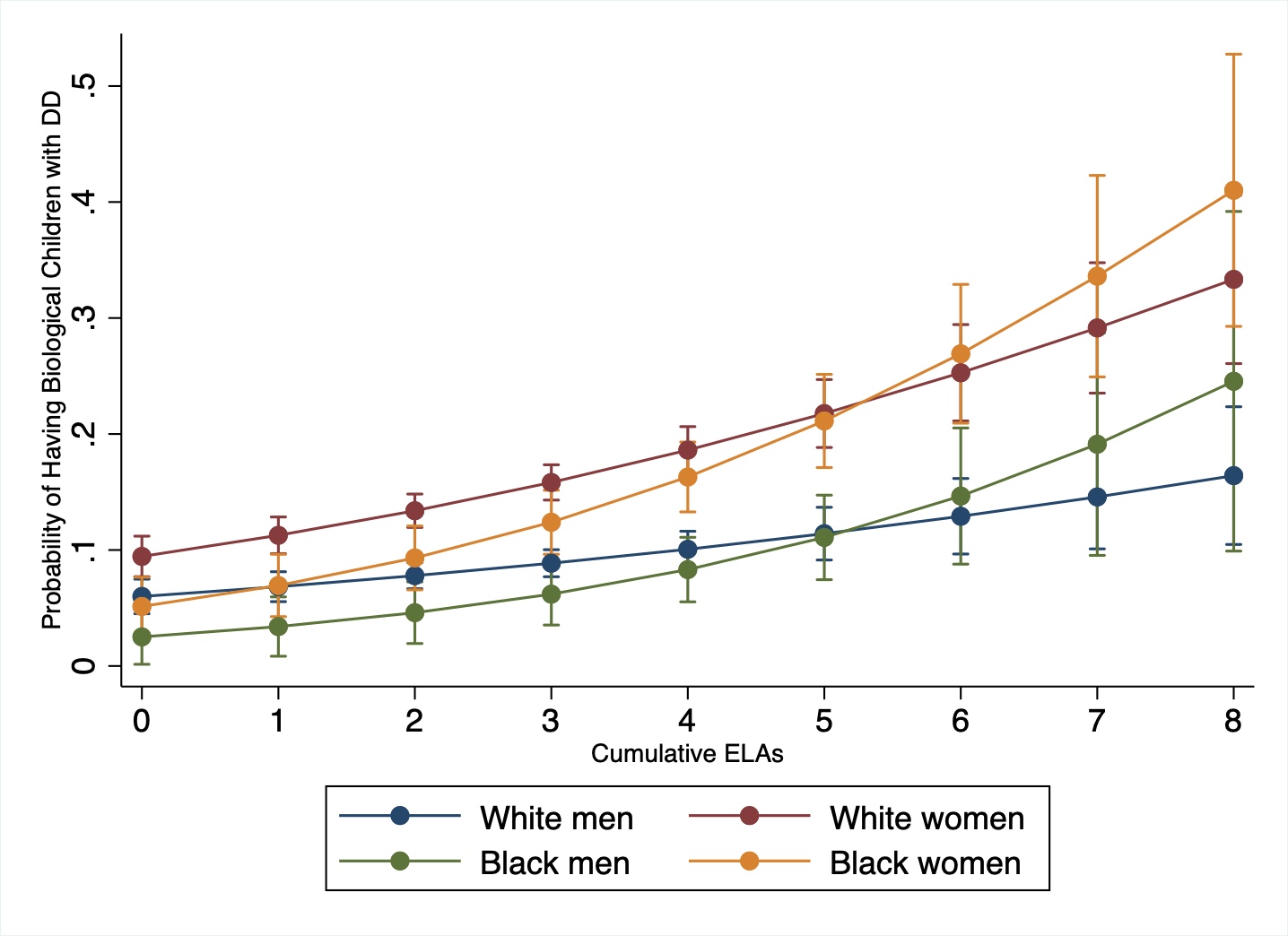

Supplement: Supplementary file 2 [file Image_1.jpg]
